# The Comparative Value of Radiation Therapy in Dermatology

**Published:** 1928

**Authors:** Ernest Dore

**Affiliations:** Physician for Skin Diseases to St. Thomas's, the Westminster and St. John's Hospitals


The Bristol
Medico-Chirurgical Journal

*"Scire est nescire, nisi id me
Scire alius sciret."*

SUMMER, 1928.

### THE COMPARATIVE VALUE OF RADIATION THERAPY IN DERMATOLOGY.

BY

ERNEST DORE, M.D., F.R.C.P.,

*Physician for Skin Diseases to St. Thomas's, the Westminster and St. John's Hospitals.*

As it would be impossible to deal exhaustively with such a comprehensive subject as that indicated by the title of this paper, I propose to discuss some general considerations first, and then refer to the cutaneous affections to which, in my experience, one or other group of radiations are especially adapted for treatment. I would remind you that we have the choice of a long scale of electro-magnetic vibrations, of which I show you a diagrammatic representation[1] ranging from the enormously long Hertzian waves representing about eleven octaves, and familiar for their use in wireless

[1] I am indebted for the loan of these slides to Messrs. John Bell and Croyden, incorporating Arnold and Son.

telegraphy, which have, as far as I know, been used only tentatively for therapeutic purposes. Then come shorter Hertzian waves, about seventeen octaves, the infra red, nine octaves, the luminous rays of the spectrum discovered as long ago as 1666 by Sir Isaac Newton, and representing only one octave on the scale, followed by the ultra-violet radiations which were mapped out into standard units by A. J. Ångström in 1868. This standard of wave length is now in general use, one unit representing a ten millionth of a millimetre. The ultra-violet portion of the spectrum may be conveniently classed as near (the longer vibrations) or far (the shorter vibrations) according to their wave lengths, the near or relatively long wave region extending from 4,000 A.U. to 3,000 A.U., and the far or shorter wave lengths from 3,000 to 1,800 A.U. As the rays become shorter they are more easily absorbed, and only penetrate the most superficial layers of the skin: according to Russ and Browning to a depth of ·1 mm. for the shorter wave lengths and less than 1 mm. for the longer wave lengths, while as they become shorter they gain in bactericidal power. Finally, the short ultra-violet radiations merge into the longer and shorter X-rays through a region which has only recently been explored by physicists, and then into the gamma rays of radium and radio-active substances. Beyond these are the little-known ultra-gamma or cosmic rays described by Professor Millikan.

The large selection we have in the employment of such of these rays as are known to produce therapeutic effects—and the latter are only known for a comparatively small group of radiations—thus becomes evident. Some day it may be possible to prescribe treatment in wave lengths, but at the present time we can only make use of certain somewhat crude sources of radiation, such as electric arcs and X-ray tubes and radium salts. Excluding the ultra-violet rays of the sun, which (according to Sir Henry Gauvain) only represent 1 per cent. of the total, the chief sources of ultra-violet light in therapeutics—since heliotherapy in this country is almost impracticable—are the carbon, tungsten and mercury vapour arcs. The choice of these depends upon the facilities of the operator as well as upon the requirements of the patient, and their relative merits have given rise to much discussion. The carbon arc light possesses longer ultra-violet radiations, and more luminous and heat rays, and necessitates longer exposures, although a larger number of patients can be treated at one time; they are chiefly used for general applications. The tungsten and mercury vapour lamps have a higher percentage of ultra-violet radiations producing quicker reaction, shorter applications being required, and are more convenient for local treatment.

I have tried all these sources of light, and, speaking for local irradiation alone, my impression is that the nature of the source of light employed, provided it be efficient, is not of great moment. Without denying the remarkable effect of actinotherapy on rickets, surgical tuberculosis, marasmus, etc., I am inclined to think that the effects of local irradiation are chiefly due to the inflammatory reaction produced in the tissues, and that this reaction has much the same effect whatever source of light be used. In other words, we have a safe, convenient and easily-controlled method of producing counter-irritation which can be employed without risk, and which has consequently become increasingly popular with the public as well as the medical profession. This comparative freedom from risk and simplicity of management is not an unmixed blessing, because it has led to extravagant statements, not only in the lay press but also by medical men who lack the experience to assess the true value of actinotherapy in special conditions with whose normal course and response to other remedies they are unfamiliar. May I say that it is only the dermatologist, or at least someone experienced in the subject, who is properly qualified to estimate the effect of any special treatment on certain cutaneous affections, such as lupus or ringworm of the scalp. I have repeatedly known cure claimed in lupus vulgaris when the ulceration has healed, and in ringworm when the scales have been removed, the underlying disease still remaining. In the same way the ophthalmic surgeon or the gynæcologist is best able to adjudicate upon diseases of special organs of which he has made a life study. In my opinion the indiscriminate use of actinotherapy by unqualified persons and by the public, who instal lamps in their bathrooms with little knowledge of how to use them, and the exaggerated claims often made for the results, are much to be deprecated. The skin and its nervous mechanism may be regarded as a receiving station which can only register certain impressions, and to expose patients to lamps containing few, if any, active rays or to various coloured lights, as is sometimes done, is about as useful as sitting them stripped in front of a loud-speaker.

*Biological Effect of Ultra-Violet Light.*—We do not even know how actinic rays act on the skin. It is generally thought that they convert the cholesterol or ergosterol of the tissues into vitamin "D," which stimulates calcium and phosphorus metabolism, and that vaso-dilatation is caused by substances absorbed as the result of changes brought about in the epithelial cells. It is hardly credible, however, that the immediate changes can be due to the local production of vitamin "D." If it is true that the rays only penetrate ·1 mm., the epidermis being avascular, it seems to me that the effect must be on the nerve endings in the superficial layers overlying the blood vessels rather than directly on the vessels themselves or on the blood or the cholesterol of the tissues.[1] It is difficult, however, to account for the erythema and pigmentation and the leucocytosis as well as the increased bactericidal power of the blood on these grounds. I have suggested elsewhere that the general effects might be due, in part, to the inhalation of ionised air, and my colleague, Dr. Allchin, exposed, at my request, a series of patients to carbon arc-light, with their bodies and faces covered, but without any definite result.

Sonne attributes much of the effect to the heat rays; but the black races, who are exposed to tropical heat and are protected by their pigment from the actinic rays, do not, so far as I am aware, differ very greatly in health from those who live in cold and sunless climates. Possibly some of the value attributed to light is in reality due to fresh air, which was shown by Leonard Hill to explain the increase of basal metabolic rate in patients undergoing light treatment. Before we contemplate installing artificial sunlight in factories and houses, might it not be equally beneficial to lay on a fresh air as well as a fresh water supply from taps? Man can live without light, but he cannot exist without air.

[1] Since writing this paper I see that Drs. McKenzie and King in their book, *Practical Ultra-Violet Therapy*, give this explanation of the effect of ultra-violet light.

Another point of interest is the effect of ultra-violet light on animals, such as that on monkeys, horses and dogs, whose hair-covered bodies should exclude the effective rays. I may mention here that, although normal hair does not fluoresce under ultra-violet light, the use of Wood's glass is employed for the detection of ringworm hairs on the scalp, although I doubt if this is an effective substitute for careful clinical examination. I have recently seen two boys from a ringworm infected school in whom the presence of a *small* quantity of grease had falsely given rise to the suspicion of infection. Now that vitamin "D" is being prepared commercially from ergosterol, it seems probable that food products will, to a certain extent, supersede the use of general ultra-violet radiation. In other words, instead of visiting the Alpine snows or erecting solaria or installing lamps for our ultra-violet rays, the same purpose may be served in the near future by swallowing a few tablets of concentrated vitamins.

*Biological Effect of X-rays.*—When we come to consider the effect of X-rays—although I do not propose to go into this in detail—there appears to be no doubt that they have a specific action on young, rapidly-multiplying cells, and their power of penetration is, of course, much greater than the longest actinic rays. Unlike the latter, they cannot be easily focused or polarised, and the latent period following their application is much longer, while harmful effects may occur in the blood and tissues long after exposure. Where actinotherapy can be administered with little risk of harm, even in unskilled hands, radio-therapy unskilfully applied has possibilities of causing serious damage to the tissues, the results often being insidious in their onset, far-reaching in their effects, and sometimes delayed for many years. If there can be any advantage in this power for harm it is that unqualified persons are, for the most part, deterred from using such a dangerous remedy, although I regret to say I have seen several patients who had been treated at an institution using rays for hypertrichosis which are said not to be X-rays, in whom serious disfigurement to the skin has resulted, the law apparently being powerless to interfere when the sufferers shrink from the publicity of taking legal action.

*Effect of Radium Rays.*—The gamma rays of radium have very much the same effect as X-rays, although they have greater powers of penetration, but the quantities obtainable and the area of application being so small, their therapeutic application is somewhat restricted, and the dangers correspondingly minimised. Having worked with these three therapeutic agents for a great number of years, I do not hesitate to say that X-rays are much the most valuable in dermatological therapeutics, and as a matter of personal experience, I may say that for one application of the ultra-violet lamp in private practice I use radium perhaps five and X-rays fifty times.

*Therapeutics of Ultra-Violet Radiation.*—Turning to the results of treatment, and referring first to the cutaneous diseases most amenable to ultra-violet light radiations, the most notable example is, of course, lupus vulgaris. This is the disease to which Finsen devoted the greater part of his attention in 1893 and 1894, and it is still being treated by concentrated arc-light at the Finsen Light Institute in Copenhagen and at the London and other hospitals. To me the light treatment of lupus vulgaris has been a disappointment. When it was originally introduced statistics were published claiming 90 per cent. of cures, but, judging from my own results of cases treated in 1900 to the present day, I doubt if such figures as these would hold good if the patients treated in the early years were produced now. I venture to think the percentage would be very much reduced. I will not, however, press this too far, as having made some remarks of a similar character last summer, I have since been receiving letters from makers of ultra-violet lamps offering to send representatives to see if my apparatus required renovating! The old Finsen treatment, with concentrated carbon light and the newer local contact applications of the mercury vapour lamp, is excellent, both in being a conservative method and in its cosmetic results, but it is tedious and slow in the extreme, complete eradication of the disease being in my experience a rare phenomenon. There is hope, however, of better results now that general light baths are being combined with local irradiation, and although I am not much impressed by the cases I have had treated in London, I believe excellent results are obtained at Leysin and at Alton and Hayling Island from heliotherapy. Other diseases in which good results are claimed are alopecia and alopecia areata, some cases of streptococcal and staphylococcal infections, certain conditions associated with acro-asphyxia, hypertrophic scars and tuberculous and septic ulcers and septic sinuses and wounds. This is not a very long list, but perhaps you will be able to supplement it from your own experience.

*Therapeutic Results of X-rays.*—When we come to X-rays the scope is much wider, and it may almost be said that there are few cutaneous diseases which cannot be treated successfully by means of appropriate doses. I shall only refer, however, to a few conditions in which X-rays take precedence over ultra-violet rays and radium. I do not propose to deal with the deep-seated X-ray therapy of malignant and other tumours, but one form of malignant growth in which the skin is chiefly involved, and which is eminently amenable to X-ray treatment, is mycosis fungoides. In the majority of cases the tumour formations disappear almost magically after small doses, and although relapses occur and the disease may spread with too great rapidity to be controlled by reasonably frequent exposures, I have treated several cases where, I believe, life has been prolonged for many years by this method. In chronic localised psoriasis two or more half-Sabouraud doses are generally sufficient to remove the patches, but the condition tends to recur, as it does after other methods of treatment, and X-ray treatment is not advisable in extensive or acute cases, although I think the results are more reliable than those claimed for actino-therapy.

Little is known of the action of X-rays on mycosis or psoriasis, but no doubt they prevent the rapid growth and multiplication of cells which occurs in these diseases possibly by altering intercellular tension. This is only a suggestion which might apply, not only to psoriasis, where there is an overgrowth of cells in an upward direction, but also to epithelioma, in which the cells grow downwards into the underlying tissues. The desiccating action of X-rays is one which I have never seen noted or explained, and might have some effects in modifying cell-tension.

In small rodent ulcers in which radium is the method of choice X-rays may prove equally useful, but in extensive ulcers they are more valuable than the former on account of the larger area that can be treated and the greater ease of application. In ulcerative and proliferative lupus and scrofulodermia a short course of X-ray treatment is valuable, and although the dangers of continued X-ray treatment in lupus are now well known, and cannot be over-emphasised, we need not be deterred from using judicious applications for short periods in properly measured and spaced doses. In tuberculous and other ulcers X-rays have a remarkably healing effect, in spite of the statements sometimes made that there is no such thing as a stimulating dose. In acne vulgaris X-rays give much better results in my experience than vaccines, although great care must be exercised in treating the face. In many cases of eczema and eczematoid conditions, staphylococcal and streptococcal affections, lichen planus, lichenification and localised pruritus, X-rays often succeed where all other methods have failed, and in the last two affections may be considered almost specific. The depilatory effect, so dangerous in the treatment of hypertrichosis, and so valuable in scalp and beard ringworm (the thallium acetate treatment of ringworm of the scalp has on account of its unreliability not superseded X-ray epilation so far), and to a less extent in staphylococcal sycosis, is another instance of the therapeutic value of X-rays, and this by no means completes the list.

*Therapeutics of Radium Rays.*—Radium, in which we chiefly utilise the gamma rays, can be employed for many of the cutaneous affections generally treated by X-rays, but as only small areas can be dealt with, owing to the small quantities at the disposal of the practitioner, its action is necessarily restricted. In dermatological practice there are four conditions in which I have found radium especially valuable. Firstly, small rodent ulcers of the face, especially when they occur in the usual situations, viz. the inner and outer canthi and eyelids and the sides of the nose, where free excision is impossible or unsatisfactory, and when cartilage or bone is not invaded. Next for keloids, if the area affected is not too extensive, radium gives better results than any other form of treatment. It is also valuable for vascular nævi, of the cavernous type, when other methods are contraindicated: and lastly, for painful warts and corns. I might mention in detail one or two cases of the last which have come under my care, as perhaps radium is not sufficiently used for the relief of these troublesome conditions when simpler measures have proved unavailing. Although of comparatively trivial nature, they often give rise to serious results by interfering with proper exercise, and leading to malposition of the foot from defective walking, not to speak of the severe pain and tenderness to which they may give rise.

One of my first cases occurred in a young girl who was anxious to visit Cambridge for the May week dances, but was prevented by a painful corn on her foot. Twenty milligrammes of radium applied for one hour gave her complete relief from pain, and she was able to fulfil her engagement and dance without any inconvenience.

A medical man, who was an athlete, had a corn on the sole of his foot, which crippled him so much that he had had it excised more than once, but without success. Radium applied for one hour relieved the pain, and a second application produced permanent relief.

These are only two early cases, and I might mention many others which have been successfully treated. In some cases, however, especially where the warts are very numerous, X-rays are more suitable, and in very hard corns radium has occasionally proved unsuccessful, probably on account of insufficient exposures.

I think I have said enough to indicate some of the conditions which may be treated by means of radiations of varying wave lengths, but we are as yet only on the threshold of what promises to be an enormous development of the scientific and therapeutic use of electro-magnetic vibrations. That there is nothing new under the sun is an old and true saying, and yet science, by splitting up these rays which have existed since the beginning of time, is finding almost every day new applications for them. In dermatology they have already to a great extent taken the place of ointments and lotions, in medicine they are competing with drugs and other therapeutic measures, and they are, perhaps, destined to render surgical procedures crude and unnecessary.

